# The Dentists’s Memorandum

**Published:** 1862-11

**Authors:** 


THE DENTIST'S MEMORANDUM. A Book of Engagements, and Manual of Ready Reference. For 1863. By C. M. CLEVELAND, M.D., Cincinnati: BRADLEY & WEBB, Printers.

This is a book of blanks and references, designed for the use of Dentists, and to serve the same purpose with them as the "Physician's Visiting List and Memorandum Book," does to the practitioner of medicine. We think the dentists will appreciate it as a very useful work, and will patronize it accordingly.

